# Cell Type Preference of a Novel Human Derived Cell-Permeable Peptide dNP2 and TAT in Murine Splenic Immune Cells

**DOI:** 10.1371/journal.pone.0155689

**Published:** 2016-05-17

**Authors:** Sangho Lim, Jung-ah Lee, Ja-Hyun Koo, Tae Gun Kang, Sang-Jun Ha, Je-Min Choi

**Affiliations:** 1 Department of Life Science, College of Natural Sciences, Hanyang University, Seoul, 133–791, Korea; 2 Research Institute for Natural Sciences, Hanyang University, Seoul, 133–791, Korea; 3 Department of Biochemistry, College of Life Science and Biotechnology, Yonsei University, Seoul, 120–749, Korea; Seoul National University College of Pharmacy, REPUBLIC OF KOREA

## Abstract

Cell-permeable peptides (CPPs) have been widely studied as an attractive drug delivery system to deliver therapeutic macromolecules such as DNA, RNA, and protein into cells. However, its clinical application is still limited and controversial due to the lack of a complete understanding of delivery efficiency in target cells. Previously we identified and characterized the novel and superior CPP, named dNP2, and here we comparatively analyzed intracellular delivery efficiency of dNP2 and TAT in various immune cells of mouse spleen to demonstrate their cell type preference. dNP2- or TAT-conjugated fluorescent proteins were most efficiently taken up by phagocytic cells such as dendritic cells and macrophages while little protein uptake was seen by lymphocytes including T cells, B cells, and NK cells. Interestingly CD8^+^ lymphoid dendritic cells and CD62L^lo^CD44^hi^ memory like T cell subsets showed significantly better uptake efficiency *in vitro* and *in vivo* relative to other dendritic cells or T cells, respectively. In addition, activated macrophages, T cells, and B cells took up the proteins more efficiently relative to when in the resting state. Importantly, only dNP2, not TAT, shows significant intracellular protein delivery efficiency *in vivo*. Collectively, this study provides important information regarding heterogeneous intracellular delivery efficiency of CPPs such as dNP2 and TAT with cell type preference in the spleen needed for its application in phagocytic cells or activated immune cells.

## Introduction

Cell permeable peptides (CPPs), either identified from various native proteins or designed as synthetic peptides, are being utilized to deliver therapeutic macromolecules into cells *in vitro* and *in vivo* [1, 2]. Since the TAT protein from HIV was found to localize to the nucleus and cytoplasm of cultured cells without the need for transfection reagents [3], the intracellular delivery of proteins by cell permeable peptides has been intensively studied. Arginine or lysine rich cationic peptides such as TAT [4], Antp [5], VP22 [6] and R9 [7] have been used over the last few decades to deliver various macromolecular cargos including siRNA [8, 9], oligonucleotides [10], peptides [11], and transcription factors [12, 13] to alter cellular behavior and modulate disease pathogenesis as a macromolecular therapeutics. Cationic CPPs bind to negatively charged cell membrane molecules such as proteoglycans on glycoproteins or negatively charged membrane lipid molecules and trigger endocytosis by the cells to uptake the CPP-cargo complex [14, 15]. Recently, human protein derived CPPs have been identified such as VectoCell [16], Lactoferin [17], Hph-1 [18], Sim-2 [11], LPIN [19], 2IL-1a [20] to minimize possible immunogenicity and toxicity *in vivo*. However, it has been demonstrated that almost all cells are permeable to CPPs without specificity, which remains one of the major challenges to the use of CPPs as an advantageous drug delivery tool [2]. Although a significant number of clinical trials have been performed using TAT, there are as of yet no available FDA approved CPP-drugs for any human disease. Here, we hypothesized that the understanding of heterogeneous delivery efficiency by a CPP would increase the success of CPP drug development.

Recently, we identified the novel human-derived CPP named dNP2 that was efficiently delivered into primary immune cells including T cells and was even localized to brain tissue through the blood-brain barrier (BBB) in mice [21]. dNP2 contains amphiphilic amino acids with basic sequences and hydrophobic amino acids with helix-loop-helix structure as prediction that seem to enhance cellular uptake by endocytosis. To better use this attractive CPP for valuable drug development, the preferential targeting of dNP2 to specific cells should be investigated. Therefore, the present study was designed to determine the cell type preference of dNP2 and TAT in various immune cells of mouse spleen. Enhanced green fluorescent proteins (EGFPs) fused to CPPs were used to treat splenocytes *in vitro* and flow cytometric analyses defined the specific cell types targeted by each CPP protein. In addition, intravenous administration of dNP2-EGFP and TAT-EGFP proteins in mice confirmed the *in vivo* relevance of heterogeneous delivery efficiency among the immune cells. Here we found that phagocytic cells such as DCs and macrophages uptake CPP-proteins more efficiently than lymphocytes. In addition, activated T cells, B cells, and macrophages are better targeted by CPP then these cells in their resting state. In addition, *in vivo* relevance suggests that there is obvious cell type preference for CPPs with heterogeneous delivery efficiency among various cell population, which should be considered for therapeutic drug design.

## Methods

### Cell lines and cell culture

EL4 (mouse lymphoma cell line) and Jurkat (human lymphoma cell line) cells were purchased from the American Type Culture Collection (ATCC) and cultured using Roswell Park Memorial Institute (RPMI) 1640 media with 10% fetal bovine serum (FBS) and 1% penicillin/streptomycin antibiotics. All cells were maintained in a 5% CO_2_ incubator at 37stitute (RPMI) 1640 media with 10% fetal bovine seThermo Scientific Hyclone.

### Purification of recombinant proteins

*Escherichia coli* BL21 (DE3) Star pLysS bacterial cells were transformed with CPP-conjugated fluorescent proteins containing pRSET-b vectors and incubated in 50 ml Lysogeny broth (LB) containing ampicillin for 12 h at 37l and shaking at 200 rpm. The cultures were transferred to 500 ml of LB media without antibiotics and incubated under the same conditions for another 1–2 h until the optical density at 600 nm (OD_600_) reached 0.4–0.6. The protein production was induced using 0.2 mM isopropyl-beta-D-thiogalactopyranoside (IPTG) for 12 h at 20°C with shaking at 150 rpm. The cells were then harvested by centrifugation at 4arv 5,000 rpm for 15 min and sonicated using the Vibra-cell VCX-130 ultrasonic processor. The supernatants were filtered through a 0.45 μM cellulose nitrate syringe filter. The 6-His tag proteins were purified through Ni-NTA affinity chromatography, and then desalted and buffer exchanged to PBS containing 10% glycerol using a PD-10 size exclusion column.

### Mice

Wild type C57BL/6 mice were purchased from Orient Bio Inc. and bred. All mice were housed and maintained in a specific pathogen-free animal facility at Hanyang University. All animal experiments were approved by the Animal Experimentation Ethics Committee of Hanyang University (Permit Number: 2015–0004, 2015–0006) and were performed in strict accordance with the guidelines of Institutional Animal Care and Use Committees of Hanyang University. And all animal research protocols followed the guidelines of Korean Association for Laboratory Animal Sciences (KALAS). All surgery was performed after CO_2_ asphyxiation euthanasia protocol and all efforts were made to minimize suffering.

### Splenocyte isolation

Spleens were removed from 6 to 10-week-old mice and chopped with scissors. The chopped tissue was incubated with 2 mg/ml collagenase D at room temperature for 20 min and red blood cells were depleted by incubating with RBC lysis buffer for 2 min. After centrifugation, the cells were resuspended in 10% FBS and 1% penicillin/streptomycin containing RPMI media and counted.

### Flow cytometric analysis of CPP-proteins delivery efficiency of *in vitro*

To analyze the delivery efficiency of CPP-conjugated proteins or CPP-peptide, 5 μM of each molecule were added to the cells and incubated for 1 h. We used 2.5X10^5^ cells/well for Jurkat cells and EL4 cells compared with splenocytes and human PBMCs, and 8X10^6^ cells/well for analyzing various splenocyte cell types. Following incubation, cells were washed twice with PBS and trypsinized with 0.25% (for cell line) or 0.05% (for primary cells) trypsin-EDTA at 37°C for 10 min. Cells were washed with PBS and stained with fluorochrome-conjugated cell surface marker specific antibodies and the intracellular delivery efficiency was analyzed using flow cytometry.

### Surface staining for flow cytometric analysis

After incubation with CPP-conjugated molecules, the cells were resuspended in 100 μl antibody mixture diluted in PBS and incubated at 4°C for 15 min. To discriminate each cell subset from the mixed cell population, different antibody combinations were used. For cell type analysis, anti-mouse CD4-PerCP-Cy5.5 (BioLegend, Clone:RM4-5), anti-mouse CD19-PE-Cy7 (BioLegend, Clone:6D5), anti-mouse NK1.1-APC (BioLegend, Clone:PK136), anti-mouse CD11b-PE-Cy7 (eBioscience, Clone:H57-597), anti-mouse CD11c-APC (eBioscience, Clone:N418), anti-mouse I-A/I-E-APC-Cy7 (BioLegend, Clone:M5/114.15.2), and anti-mouse F4/80-PerCP-Cy5.5 (BioLegend, Clone:BM8) were used. Also, anti-mouse CD16/32 (BioLegend, Clone:93) was used for blocking the Fc receptor. For activated T cell analysis, anti-mouse CD69-FITC (BioLegend, Clone:H1.2F3), anti-mouse CD4-APC (eBioscience, Clone:GK1.5), and anti-mouse CD8-PerCP-Cy5.5 (eBioscience, Clone:53–6.7) were used. For activated B cell analysis, anti-mouse CD86-PE-Cy5 (BioLegend, Clone:GL-1), anti-mouse IgG2a, kappa-isotype (BioLegend, Clone:RTK2758), and anti-mouse CD19-PE (BioLegend, Clone:6D5) were used.

### Immune cell activation

To activate splenic T cells, anti-mouse CD3 antibody (2 μg/ml, BD Pharmingen) was coated onto 96-well plates (BD Falcon) at 3796-well plateellmousesplenocytes were seeded at a density of 2.5X10^5^ cells/well and incubated overnight at 37 r in a 5% CO_2_ incubator. To activate B cells, DCs, or macrophages, isolated splenocytes were cultured in 12-well plates at 4X10^6^ cells/well with 5 μg/ml (for B cells) or 100 ng/ml (for DCs and macrophages) of *Escherichia coli* 055:B5 (Sigma-Aldrich) in each well and incubated overnight at 37°C in 5% CO_2_ incubator.

### Flow cytometric analysis of CPP-protein delivery efficiency *in vivo*

5 mg of EGFP, CPP-EGFP, or PBS was intravenously injected into 6-week-old C57BL/6 female mice. After 1 h, the mice were sacrificed and spleens were removed for isolating splenocytes from a single cell suspension. The cells were stained with fluorochrome-conjugated surface marker specific antibodies and analyzed via flow cytometry.

### Statistical analysis

The data were analyzed using one-way ANOVA, two-way ANOVA with multiple comparison tests or Student’s *t-*test. *p* values <0.05 were considered significant. Statistical analysis was performed using Prism 6 (Graphad Software, Inc.).

## Results

### dNP2- and TAT-proteins are delivered more efficiently to phagocytic cells than lymphocytes

**Fig 1:**
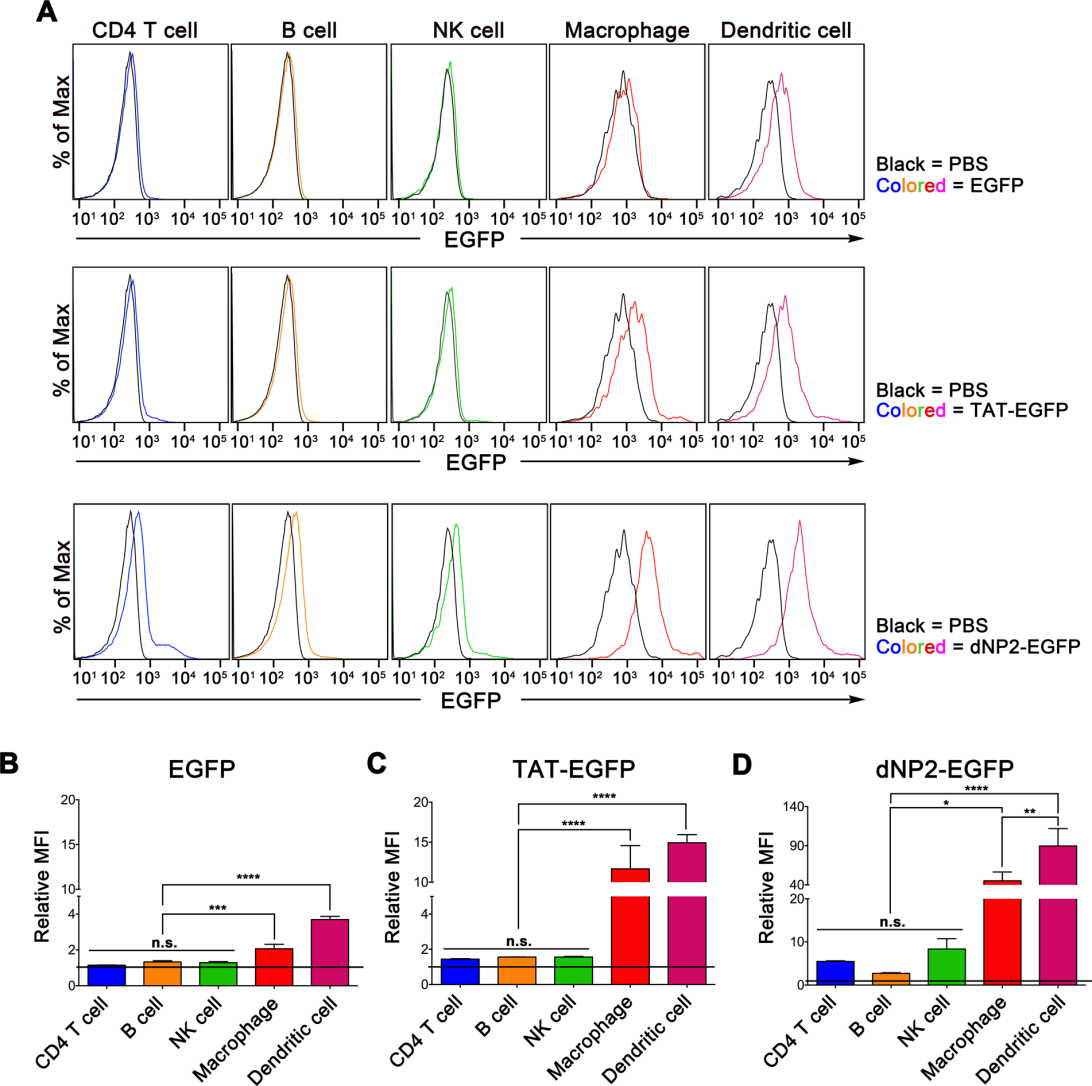
Protein delivery efficiency of dNP2 and TAT in various immune cells. (A) 5 μM of EGFP, TAT-EGFP, dNP2-EGFP, or PBS were treated to splenocytes from 6-week-old C57BL/6 mice and protein delivery efficiencies were analyzed by flow cytometry after cell staining with cell type specific markers for CD4^+^ CD4 T cells, CD19^+^ B cells, NK1.1^+^ NK cells, CD11c^low^CD11b^high^F4/80^+^ macrophages, and MHCII^+^CD11c^high^ DCs. (B-D) The experiments were independently repeated three times and are presented as the relative mean fluorescence intensity (relative MFI). The values were normalized with the MFI of PBS treated samples. Bar graphs indicate the mean ± s.d. One-way ANOVA was used for statistical analysis and * indicates p<0.05, ** indicates p<0.01, *** indicates p<0.001, **** indicates p<0.0001.

To determine cell type heterogeneity of dNP2-protein delivery efficiency in various immune cells of mouse spleen, we treated dNP2-EGFP or TAT-EGFP to splenocytes and intracellular delivery efficiency was analyzed by flow cytometry by the staining of cell type specific markers (Fig 1A–1D). The relative mean fluorescent intensity (Relative MFI) represents fold increase that each MFI is normalized to PBS treated sample (Fig 1B–1D). Statistical significance among the all groups were comparatively analyzed by one-way ANOVA test with Sidak’s multiple comparison test (S1 Fig). Both dNP2-EGFP and TAT-EGFP proteins were delivered into CD11c^lo^CD11b^hi^F4/80^+^ macrophages and CD11c^+^MHCII^+^ DCs, which are phagocytic and antigen presenting cells, with significantly higher efficiency than into lymphocytes. In the case of CD4 T cells, B cells, and NK cells, only dNP2-EGFP showed significant protein delivery efficiency comparing with PBS control while TAT-EGFP did not at 5 μM. This pattern is almost identical at various incubating time points including 10 min, 30 min, 1 h, 2 h or 6 h (S2 Fig) and also consistent in the FACS-sorted each cell population (S3 Fig). Interestingly, even EGFP protein without CPPs was significantly taken up by macrophages as well as DCs although their relative MFI value is much lower than that of dNP2- or TAT-EGFP treated group (Fig 1B). These results collectively suggest that phagocytic cells such as macrophages and DCs uptake CPP-protein more efficiently than lymphocytes, and CPPs such as dNP2 and TAT could significantly deliver a cargo protein into those cells to modulate innate immunity including antigen presentation, inflammatory response, etc.

### CD8^+^ dendritic cells are the major cell type to uptake dNP2- and TAT-proteins

**Fig 2:**
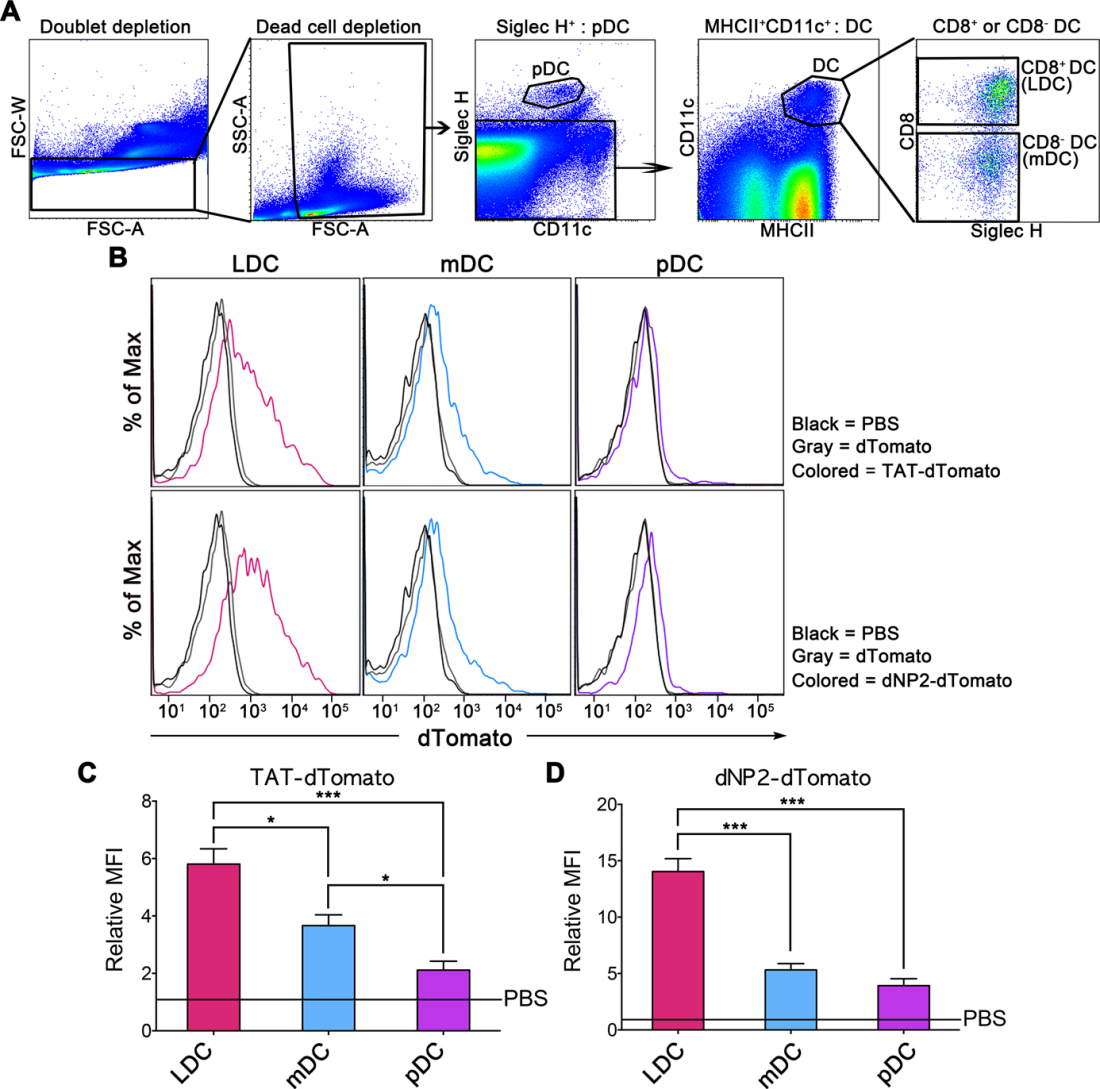
Protein delivery efficiency of dNP2 and TAT among dendritic cell subsets. 5 μM dTomato, TAT-dTomato, dNP2-dTomato, or PBS were treated to splenocytes from 6-week-old C57BL/6 mice and the cells were analyzed by flow cytometry. (A) The cells were subdivided into Siglec H^-^CD11c^+^MHCII^+^CD8^+^ lymphoid CD8^+^ DCs, Siglec H^-^CD11c^+^MHCII^+^CD8^-^ myeloid CD8^-^ DCs, and Siglec H^+^ pDCs. (B) The protein delivery efficiencies into each subset of DCs were analyzed by flow cytometry. (C-D) The experiments were independently repeated three times and are presented as the relative mean fluorescence intensity (relative MFI). The values were normalized with the MFI of PBS treated samples. Bar graphs indicate the mean ± s.d. and * indicates *p*<0.01 and *** indicates *p*<0.0001 from one-way ANOVA with Tukey’s multiple comparison test (n = 3, (C) F = 59.46, P<0.0001 and (D) F = 134.5, P<0.0001).

Splenic DCs can be divided into subsets including lymphoid CD8^+^ DCs, myeloid CD8^-^ DCs, and plasmacytoid DCs (pDCs) based on different biological characteristics [22–24]. To determine CPP-protein delivery efficiency in splenic DC subsets, splenocytes were treated with red fluorescent dTomato protein fused with dNP2 or TAT (dNP2-dTomato or TAT-dTomato). Intracellular red fluorescence intensity and each DC subset marker were analyzed by flow cytometry. First, we determined the DC subsets as Siglec H^+^ pDC, Siglec H^-^CD11c^+^MHCII^+^CD8^+^ lymphoid CD8^+^ DC, and Siglec H^-^CD11c^+^MHCII^+^CD8^-^ myeloid CD8^-^ DC (Fig 2A). Both TAT-dTomato and dNP2-dTomato proteins were delivered into the CD8^+^ DCs with significantly higher efficiency relative to CD8^-^ DCs. In addition, pDCs did not uptake CPP-proteins suggesting that the major cellular subset to uptake CPP-proteins is CD8+ lymphoid DCs (Fig 2B, 2C and 2D). These results suggest that among the DC subsets, CPP-protein uptake efficiency is quite variable even in DC subsets and that lymphoid DCs have higher delivery efficiency than myeloid DCs and pDCs indicating that immunization with CPP-conjugated antigen cargos would be efficient application using CPPs such as dNP2 and TAT. mDCs also showed significantly higher delivery efficiency than pDCs (Fig 2C and 2D) suggesting that targeting mDCs by CPP-mediated cargo delivery would be good approaches such as anti-inflammatory functions.

### Activated lymphocytes uptake dNP2- and TAT-proteins more efficiently than resting state lymphocytes

**Fig 3:**
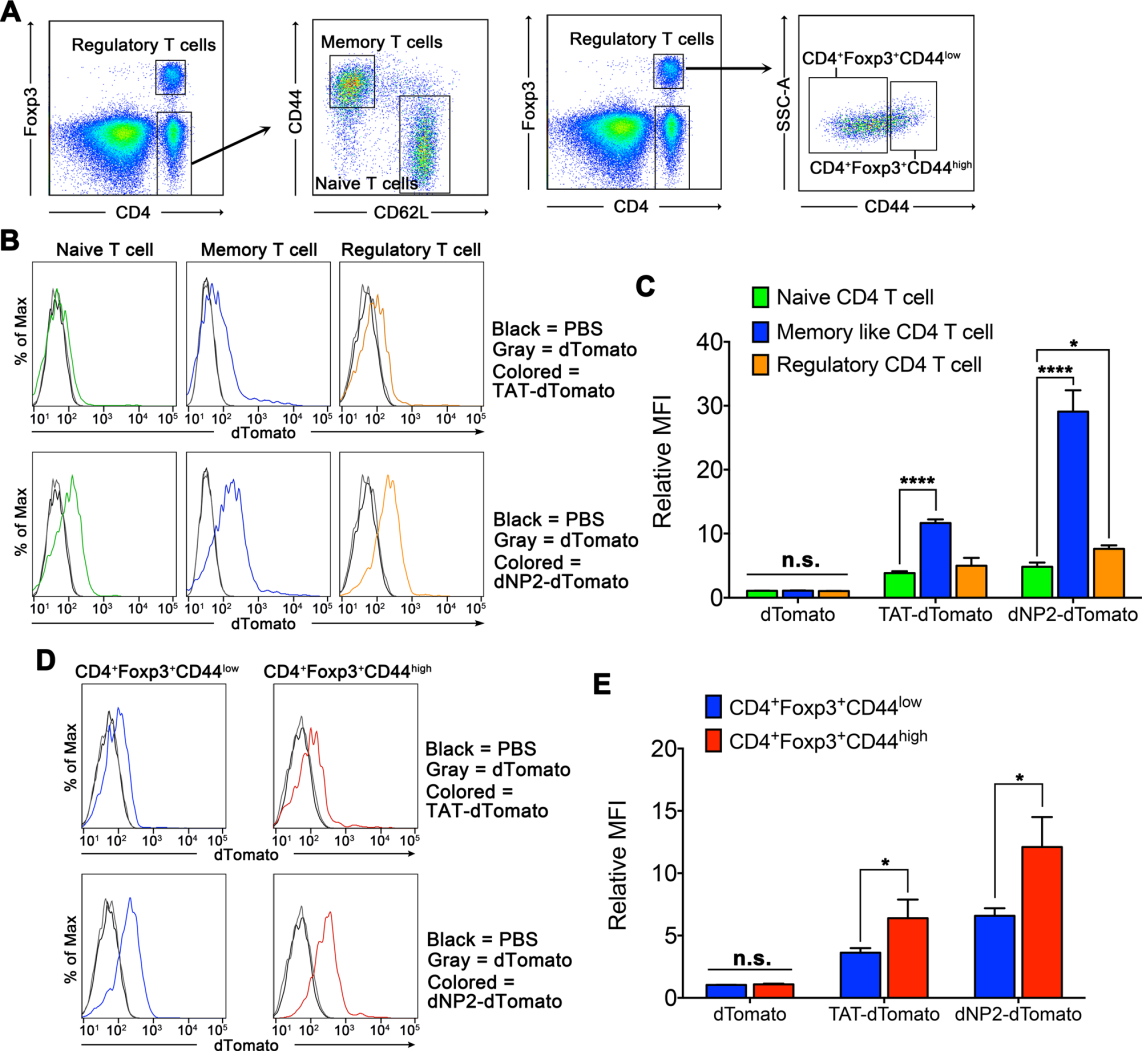
Protein delivery efficiency of dNP2 and TAT on naïve, regulatory, or memory-like CD4 T cells. 5 μM dTomato, TAT-dTomato, dNP2-dTomato or PBS were treated to splenocytes from Foxp3-GFP transgenic 6-week-old C57BL/6 mice and the cells were analyzed by flow cytometry. (A) The cells were subdivided into CD4^+^Foxp3^+^ regulatory T cells, CD4^+^CD62L^high^CD44^low^ naïve CD4 T cells, and CD4^+^CD62L^low^CD44^high^ memory-like CD4 T cells by flow cytometry after cell surface marker staining. Also regulatory T cells were subdivided into Foxp3^+^CD44^low^ and Foxp3^+^CD44^high^ cells. (B) The protein delivery efficiencies into each subset of CD4 T cells were analyzed by flow cytometry. (C) The experiments were independently repeated three times and are presented as the relative mean fluorescence intensity (relative MFI). The values were normalized with the MFI of PBS treated samples. Bar graphs indicate the mean ± s.d. and within groups (dTomato, TAT-dTomato and dNP2-dTomato) the differences of means of memory like CD4 T cells and regulatory CD4 T cells were compared with means of naïve CD4 T cells by two-way ANOVA with Dunnett’s multiple comparison test, * indicates *p<*0.05 and **** indicates *p*<0.0001 (n = 3). (D) The protein delivery efficiencies into each subset of regulatory T cells were analyzed by flow cytometry. (E) The values were normalized with the MFI of PBS treated samples. Bar graphs indicate the mean ± s.d. and the data were analyzed by student’s *t-*test, *indicates *p*<0.05 (n = 3).

Because activated lymphocytes are rapidly proliferating and their membrane proteins are dynamically regulated, we hypothesized that CPP-proteins would be delivered into activated or memory-like lymphocytes with higher efficiency relative to resting state lymphocytes. To examine this, we treated dNP2-dTomato or TAT-dTomato to mouse splenocytes. Then, we at first subdivided CD4 T cells into CD4^+^CD62L^hi^CD44^lo^ naïve CD4 T cells, CD4^+^CD62L^lo^CD44^hi^ memory-like CD4 T cells, and CD4^+^Foxp3^+^ regulatory T cells by flow cytometry and determined heterogeneous delivery efficiency by CPP-proteins (Fig 3A). Both dNP2-dTomato and TAT-dTomato protein, but not dTomato without CPPs, delivery efficiencies were significantly higher in CD62L^lo^CD44^hi^ memory like activated CD4 T cells than in naïve CD4 T cells and Foxp3^+^ regulatory T cells, though the efficiency of dNP2 is much higher than TAT (Fig 3B and 3C). Also the delivery efficiency was significantly higher in CD4^+^Foxp3^+^CD44^high^ cells than that of CD4^+^Foxp3^+^CD44^low^ cells (Fig 3D and 3E). This result suggests that among the peripheral T cell subsets, CPP-protein delivery efficiency is heterogeneous that memory-like T cells or effector T cells are preferred by CPP-protein delivery than naïve T cells.

**Fig 4:**
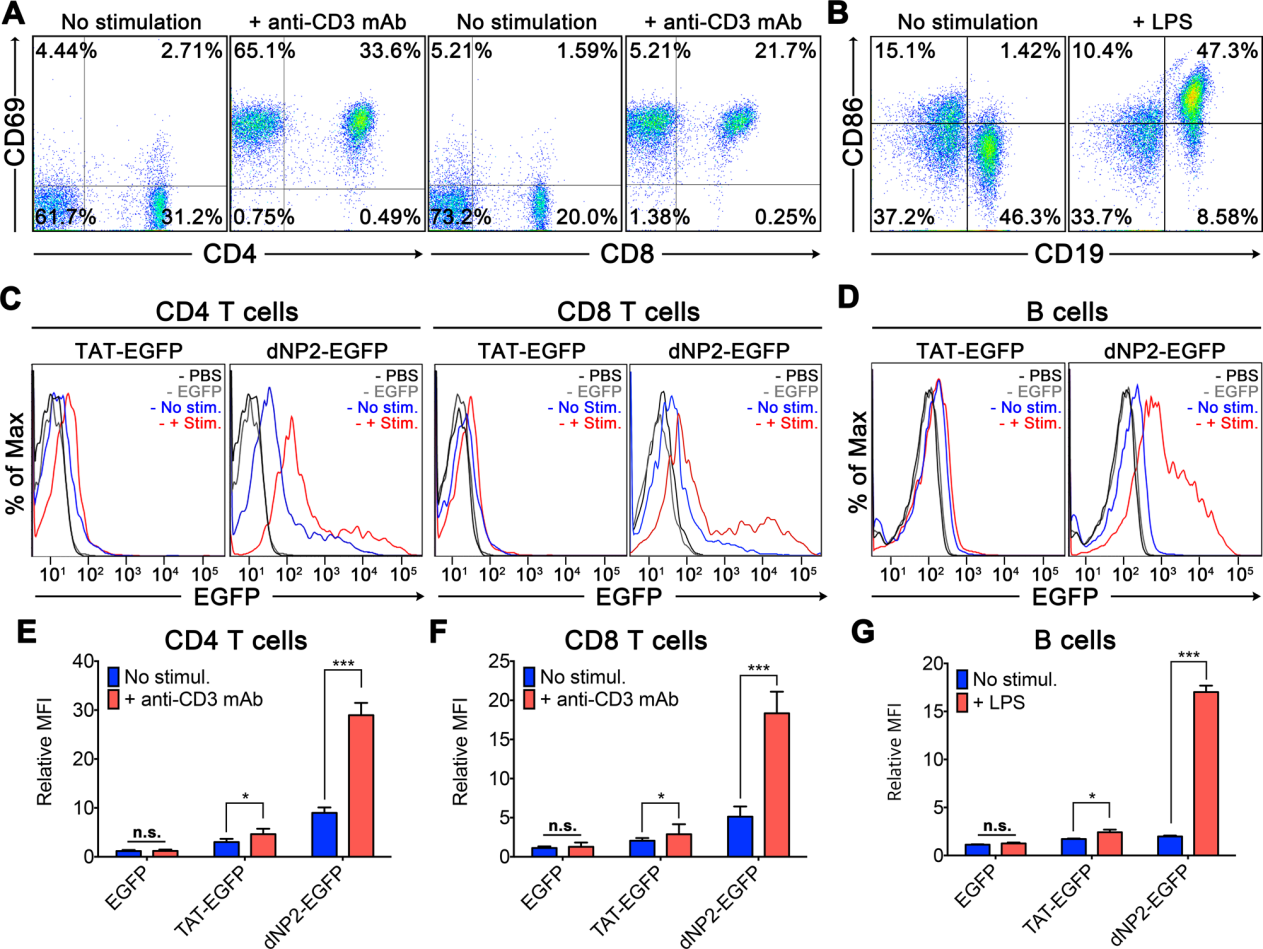
Protein delivery efficiency of dNP2 and TAT on stimulated or unstimulated T or B cells. Splenocytes from 6-week-old C57BL/6 mice were stimulated with (A) 2 μg/ml anti-mouse CD3 antibody for 12 h or (B) 5 μg/ml LPS for 24 h and additionally incubated in the presence of 5 μM EGFP, TAT-EGFP, dNP2-EGFP, or PBS for 30 min. (C) The protein delivery efficiency into stimulated or unstimulated CD4 or CD8 T cells was analyzed by flow cytometry. (D) The protein delivery efficiency into stimulated or unstimulated B cells was analyzed by flow cytometry. (E-G) The experiments were independently repeated three times and are presented as the mean fluorescence intensity (MFI). Bar graphs indicate the mean ± s.d. and * indicates *p*<0.05 and ** indicates *p*<0.01 from Student’s t-tests (n = 5).

Next, we stimulated isolated mouse splenocytes with plate bound anti-CD3 and anti-CD28 antibodies to determine whether activated CD4 and CD8 T cells would uptake CPP-proteins more efficiently relative to unstimulated cells (Fig 4A). Both dNP2-EGFP and TAT-EGFP proteins were delivered into activated CD4 and CD8 T cells with significantly higher efficiency than unstimulated cells (Fig 4C, 4E and 4F). In addition, we stimulated splenocytes with lipopolysaccharide (LPS) to examine whether activated B cells would uptake CPP-proteins (Fig 4B). From the results, LPS stimulated B cells uptake dNP2-EGFP and TAT-EGFP proteins more efficiently than resting B cells (Fig 4D and 4G). These results collectively suggest that CPPs such as dNP2 and TAT could deliver a cargo protein into activated immune cells with better efficiency than their resting state that it would be valuable to apply on modulating abnormally activated T or B cell responses in allergic or autoimmune diseases.

### Activated macrophages, but not activated dendritic cells, uptake dNP2- and TAT-proteins more efficiently than resting state

**Fig 5:**
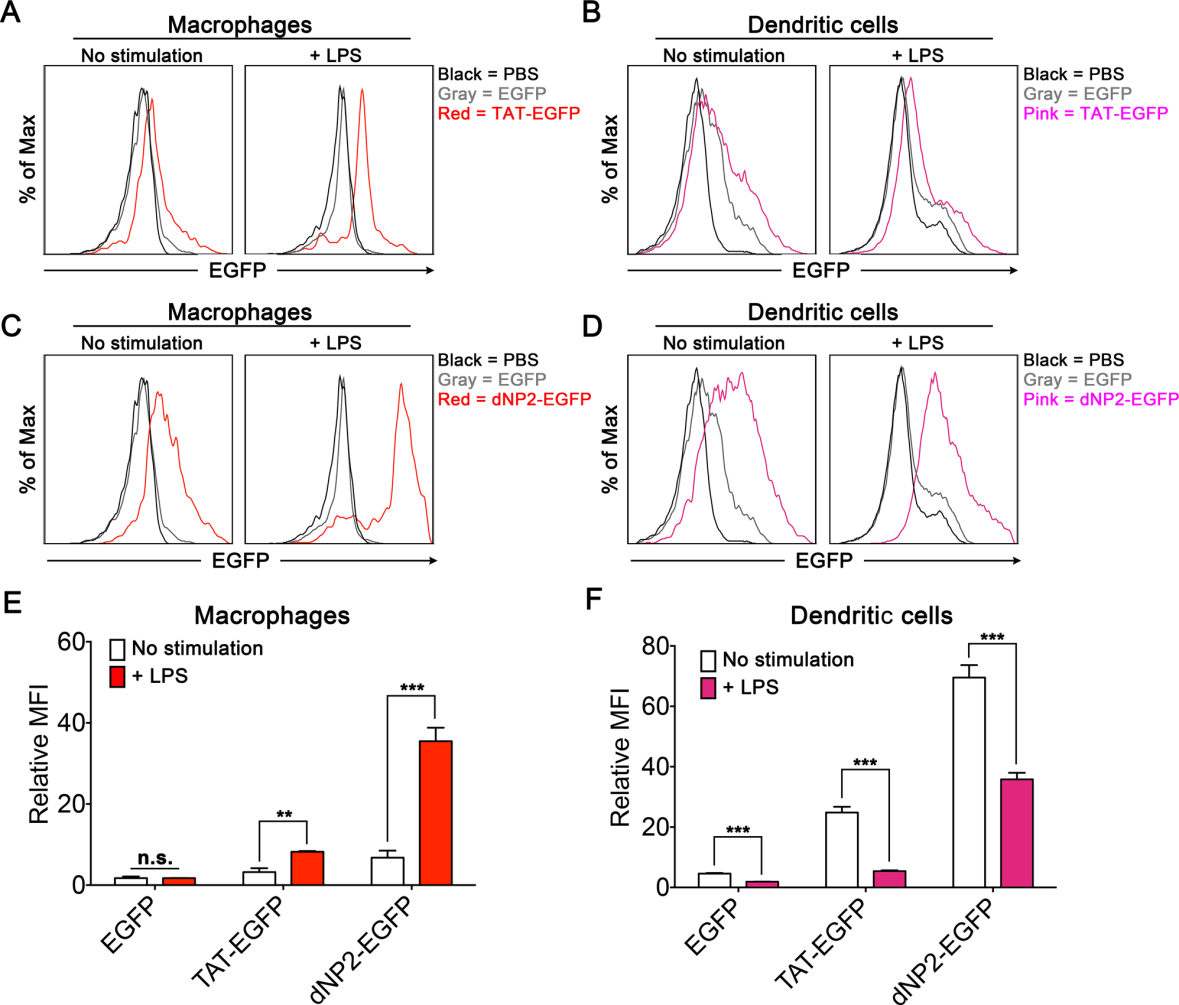
Protein delivery efficiency of dNP2 and TAT on unstimulated or LPS-stimulated macrophages or dendritic cells. Splenocytes from 6-week-old C57BL/6 mice were stimulated with 5 μg/ml LPS for 24 h and additionally incubated in the presence of 5 μM EGFP, TAT-EGFP, dNP2-EGFP, or PBS for 30 min. (A-D) The protein delivery efficiency into stimulated or unstimulated macrophages or DCs was analyzed by flow cytometry. (E-F) The experiments were independently repeated three times and are presented as the relative mean fluorescence intensity (relative MFI). The values were normalized with the MFI of PBS treated samples. Bar graphs indicate the mean ± s.d. and *** indicates *p*<0.001 from Student’s t-tests (n = 3).

We next examined CPP-protein delivery efficiency in activated macrophages and DCs compared to their resting state. Isolated mouse splenocytes were incubated with or without LPS stimulation overnight, and then cells were treated with dNP2-EGFP or TAT-EGFP protein for 1 h. The intracellular fluorescence of CD11c^lo^CD11b^hi^F4/80^+^ macrophages and CD11c^+^MHCII^+^ DCs was analyzed by flow cytometry. dNP2-EGFP treated LPS-stimulated macrophages showed significantly higher intracellular fluorescence intensity than unstimulated macrophages and TAT-EGFP treated macrophages also showed a similar delivery pattern even though the delivery efficiency of dNP2 is much higher than TAT (Fig 5A, 5C and 5E). However, CPP-protein delivery efficiency in LPS-stimulated DCs was significantly lower than in resting DCs (Fig 5B, 5D and 5F) possibly due to maturated DCs have less antigen uptake ability then immature state [25]. Interestingly EGFP without CPPs also shows consistently reduced protein uptake efficiency by DCs while no difference was observed in macrophages (Fig 5E and 5F). These results suggest that CPP-protein delivery efficiency is more significant in activated macrophages and immature DCs that it could be valuable to apply it on modulating inflammatory responses mediated by macrophages or on initiating immune responses mediated by DCs.

### dNP2- but not TAT-proteins shows consistent cell type preference in various immune cells of mouse spleen *in vivo*

**Fig 6:**
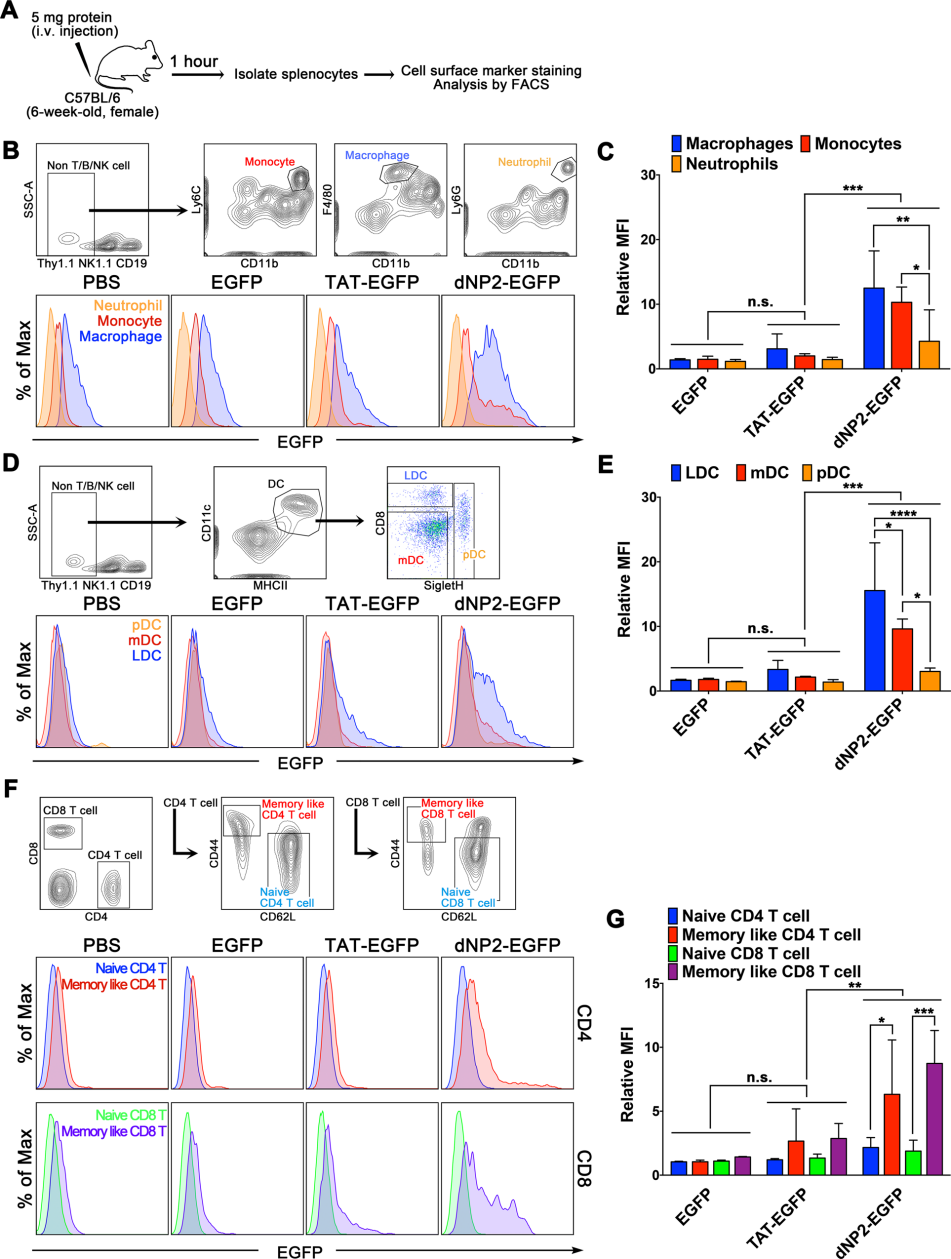
Protein delivery efficiency of dNP2 and TAT in various immune cells *in vivo*. (A) Experimental scheme is indicated. EGFP, TAT-EGFP, dNP2-EGFP, or PBS (5 mg) were injected to 6-week-old C57BL/6 mice and after 1 h the splenocytes were isolated and analyzed by flow cytometry after cell surface marker staining. (B-C) Protein delivery efficiency into Ly6C^+^CD11b^high^ monocytes (red), F4/80^high^CD11b^int^ macrophage cells (blue), and Ly6G^+^CD11b^high^ neutrophils (orange) was analyzed by flow cytometry. (D-E) Protein delivery efficiency into CD8^-^Siglec H^-^ myeloid DCs (red), CD8^+^Siglec H^-^ lymphoid DCs (blue), and Siglec H^+^ pDC (orange) was analyzed by flow cytometry. (F-G) Protein delivery efficiency into CD4^+^CD62L^high^CD44^low^, CD4^+^CD62L^low^CD44^high^, CD8^+^CD62L^high^CD44^low^, or CD8^+^CD62L^low^CD44^high^ cells was analyzed by flow cytometry. Bar graphs indicate the mean ± s.d. and within groups (EGFP, TAT-EGFP, dNP2-EGFP) the differences of means of each cell types were compared with each other by two-way ANOVA with Tukey’s multiple comparison test, * indicates *p*<0.05, ** indicates *p*<0.01, *** indicates *p*<0.001, **** indicates *p*<0.0001 (n = 3).

Finally, we investigated the applicability of *in vitro* results *in vivo* by intravenous administration of dNP2-EGFP or TAT-EGFP protein in mice. One hour after the intravenous injection of 5 mg dNP2-EGFP or TAT-EGFP into mice, splenocytes were isolated and various immune cell types were stained with cell type specific marker antibodies (Fig 6A). As consistent with the *in vitro* results, F4/80^high^CD11b^int^ macrophage cells (blue) are the most major cells to uptake proteins. In addition, delivery efficiency of dNP2-EGFP shows higher delivery efficiency on Ly6C^+^CD11b^high^ monocytes than Ly6G^+^CD11b^high^ neutrophils (Fig 6B and 6C). Among the DC subsets, CD8^+^ Siglec H^-^ lymphoid DCs (blue) showed higher delivery efficiency than CD8^-^ Siglec H^-^ myeloid DCs and Siglec H^+^ pDCs (Fig 6D and 6E). CD62L^lo^CD44^hi^ memory-like activated cells (red) in both CD4 and CD8 T cells preferentially transduced by dNP2-EGFP protein relative to CD62L^hi^CD44^lo^ naïve CD4 and CD8 T cells (Fig 6F and 6G). However, importantly, TAT-EGFP delivery efficiency was not significant in any cell type population suggesting its limitation for *in vivo* application. These results suggest that dNP2 efficiently deliver a cargo protein into various immune cells *in vivo* with heterogeneous delivery efficiency and this should be considered for its application design to modulate cellular responses *in vivo*.

## Discussion

In the present study, we characterized and demonstrated cell type preference of the recently identified human derived BBB-permeable peptide dNP2 [21] and the widely used HIV-derived CPP, TAT [4, 26] for the uptake of proteins in various immune cells. Both dNP2 and TAT conjugated fluorescent proteins were more preferentially delivered into phagocytic cells such as macrophages and DCs relative to lymphocytes. In addition, we confirmed that activated immune cells including macrophages, T cells, and B cells could uptake proteins with better efficiency relative to their resting state, collectively demonstrating heterogeneous intracellular delivery efficiency of dNP2 and TAT *in vitro* and *in vivo* should be considered for better application of these CPPs in disease modulation.

CPPs are generally regarded as a non-specific macromolecular delivery tool, which has been considered a major disadvantage of CPP usage for therapeutic purposes [27–29]. However, previous studies reported that the arginine-based CPP r8 peptide, without any targeting sequences, spontaneously accumulated in xenografted tumors of tumor-bearing nude mice [30]. Even though the R8 peptide was delivered into normal tissues, it showed a preference for tumor cells. In addition, in the early days of CPPs, Dowdy et al. reported an odd delivery pattern in spleen tissue *in vivo* where TAT-β-galactosidase was only detected in red pulp while it was clearly not detected in white pulp in mice [4]. These studies suggested that CPPs could be delivered with different efficiencies and specificities depending on target cell type. These previous results suggest the possibility of cell type preference in various types of cells *in vivo*.

In the present study, we revealed that both dNP2 and TAT have cell type preference in various immune cells. Although the proportion of DCs and macrophages are just 1–2% of total spleen cells, their CPP-protein uptake efficiency is much dominant compare to the lymphocytes including T cells or B cells. This suggests that the phagocytic action or cell’s own characteristics are important in effective CPP-protein delivery. High efficiency in phagocytic cells including DCs and macrophages also suggest that the application of CPPs such as dNP2 as an immunization tool to delivery of antigens for vaccine development could be one of the best approaches. Especially, lymphoid DCs were shown to uptake proteins much more efficiently than myeloid DCs and plasmacytoid DCs. Previously, it has been reported that lymphoid DCs have much higher T cell stimulation capacity than myeloid DCs [31]. While, myeloid DCs have been known as having higher migratory capacity compared with lymphoid DCs [32–34]. In addition, only lymphoid DC can cross-prime cytotoxic T cells *in vivo* [35]. Thus, lymphoid DCs which is preferred by CPP-cargo delivery is appropriate target for DC vaccination using cell-penetrating peptide [36]. Similar to the preference and high delivery efficiency of TAT to lymphoid DCs in this study, previous studies showed that TAT-conjugated protein antigen could be a useful DC vaccination methodology for boosting antigen specific CD8 T cell immunity [37, 38]. For example, treatment of the TAT conjugated *Leishmania* antigen TAT-LACK to DCs successfully induced *Leishmania* specific CD8 T cells *in vitro* while LACK alone could not [39]. Also, numerous studies reported that DC vaccination is one of the most attractive approaches for cancer immune therapy using specific cancer antigens [40–43]. TAT conjugated breast cancer specific antigen generated cancer specific CD8 T cells and infiltrated into tumor tissues *in vivo* [44]. Our study previously showed that dNP2 has higher delivery efficiency relative to TAT suggesting that dNP2 could be more valuable to deliver antigens for the generation of pathogen or cancer specific immunity.

Macrophages are another potent target cell type for modulating the immune system because of their high delivery efficiency with both dNP2 and TAT. Macrophages are one of the most abundant cells existing in every tissue including lymphoid, lung, liver, bone, and brain tissues with phenotypic diversity [45, 46]. For this reason, the preference of dNP2-protein delivery to macrophages could be a useful tool for modulating macrophage functions regarding inflammation, disease pathogenesis, or bone remodeling.

Both dNP2 and TAT conjugated protein were taken up by memory like CD4 T cells or activated lymphocytes including CD4 and CD8 T cells or B cells with higher efficiency relative to these cells in their resting state. This preference would have the advantage of modulating activated lymphocytes and memory T and B cells by delivering signaling modulatory molecules. Previously we have attempted to deliver cytoplasmic domain of CTLA-4 protein which inhibits T cell receptor signaling pathway in activated T cells modulated abnormal immune responses in allergic or autoimmune disease models [18, 21, 47]. In addition, CPP-conjugated Foxp3 protein could modulated OVA-induced allergic T cell responses *in vivo* without affecting the other T cells response to KLH antigen which was still intact [12]. Other previous report showed that CPP-based antisense-oligomer delivery successfully modulated T cell response by altering gene expression [48]. These results suggest CPPs such as dNP2 and TAT could be applied to modulate activated lymphocyte reaction by passive targeting or preference moiety.

In consistency of relative preference on activated T cells, regulatory T cells which is highly expressing CD44, that could be regarded as activated/effector regulatory T cell [49], showed significantly higher CPP-protein uptake efficiency than resting regulatory T cells which have lower level of CD44. Regulatory T cell subsets were reported as CD45RA^+^Foxp3^low^ resting regulatory T cells, CD45RA^-^Foxp3^high^ activated regulatory T cells and CD45RA^-^Foxp3^low^ nonsuppressive T cells [50]. In the case of carcinoma patients, the activated regulatory T cells are increased in the peripheral circulation and antitumor immunity is suppressed [51]. The CPP-drugs could be preferably delivered into activated regulatory T cells for cancer therapy which possibly be a novel approach for cancer-immunotherapy.

Collectively, we demonstrate cell type preference of CPP delivery efficiency using dNP2 and TAT in various immune cells *in vitro* and *in vivo*. First, CPP delivery efficiency is the highest into phagocytic cells, especially lymphoid DCs. Second, CPP delivery efficiency is much higher into the activated immune cells than non-activated immune cells. Last, *in vivo* protein delivery efficiency of TAT is as little as negative control suggesting significant advantage of dNP2 usage *in vivo*. These findings suggest that heterogeneity of CPP-protein delivery efficiency would be dependent on cells own character such as phagocytic activity, etc. Cell type preference analysis in non-immune cells also should be considered and it would provide important additional information for the accurate application of CPPs in appropriate target cells.

## Supporting Information

S1 Fig One-way ANOVA with Sidak’s multiple comparison test results of Fig 1B–1D. (TIF)

S2 Fig Time dependent protein delivery efficiency of CPP-EGFPs in various immune cells. 5 μM of EGFP, TAT-EGFP, dNP2-EGFP or PBS were treated to splenocytes from 6-week-old C57BL/6 mice for various reaction times including 10 min, 30 min, 1 h, 2 h or 6 h and protein delivery efficiencies were analyzed by flow cytometry after cell staining with cell type specific markers (CD4^+^ CD4 T cells, CD19^+^ B cells, NK1.1^+^ NK cells, CD11c^low^CD11b^high^F4/80^+^ macrophages, and MHCII^+^CD11c^high^ DCs). The values were normalized with the mean fluorescence intensity (MFI) of PBS treated samples (relative MFI). The graphs indicate the mean ± s.d. In the case of TAT-EGFP and dNP2-EGFP, macrophages and DCs showed significantly higher delivery efficiency than lymphocytes within every time points. We performed statistical analysis by two-way ANOVA with Tukey’s multiple comparisons test and *** indicates p<0.001 and **** indicates p<0.0001.
(TIF)

S3 Fig Protein delivery efficiency of CPP-EGFPs into FACS sorted various immune cells. 5 μM of EGFP, TAT-EGFP, dNP2-EGFP or PBS were treated to FACS sorted CD4 T cells (CD4^+^), CD8 T cells (CD8^+^), B cells (CD19^+^), Dendritic cells (MHCII^+^CD11c^high^) or Macrophages (CD11c^low^CD11b^high^F4/80^+^). The delivery efficiencies were analyzed by flow cytometry. In the bar graphs, the values were normalized with MFI of PBS treated samples (relative MFI).
(TIF)
