# Supplementary figures and images for: Cell Type Preference of a Novel Human Derived Cell-Permeable Peptide dNP2 and TAT in Murine Splenic Immune Cells
Source: PLoS One. 2016 May 17;11(5):e0155689. doi: 10.1371/journal.pone.0155689 (PMC4871486; doi:10.1371/journal.pone.0155689)

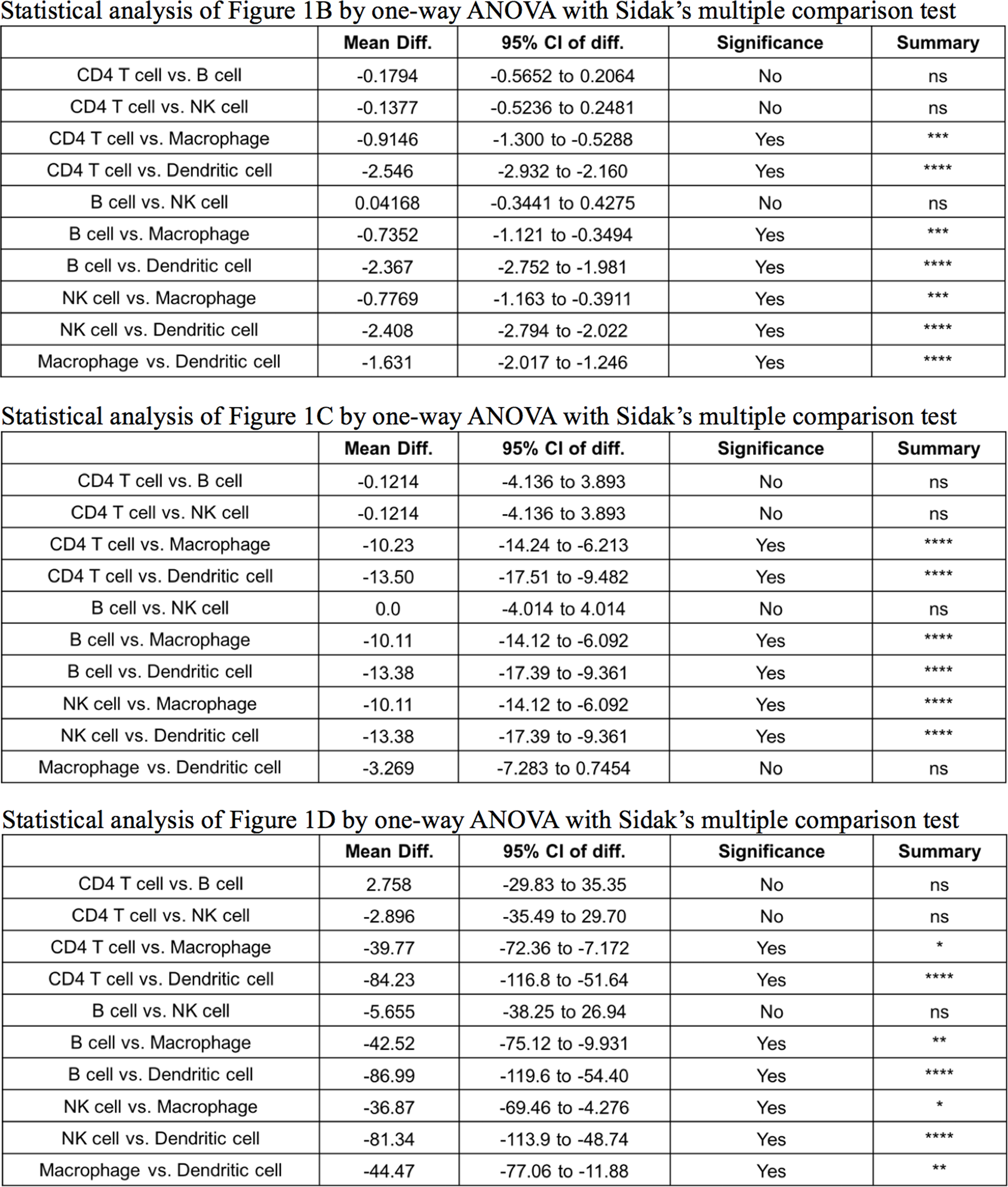

Supplement: S1 Fig — (TIF) [file pone.0155689.s001.tif]

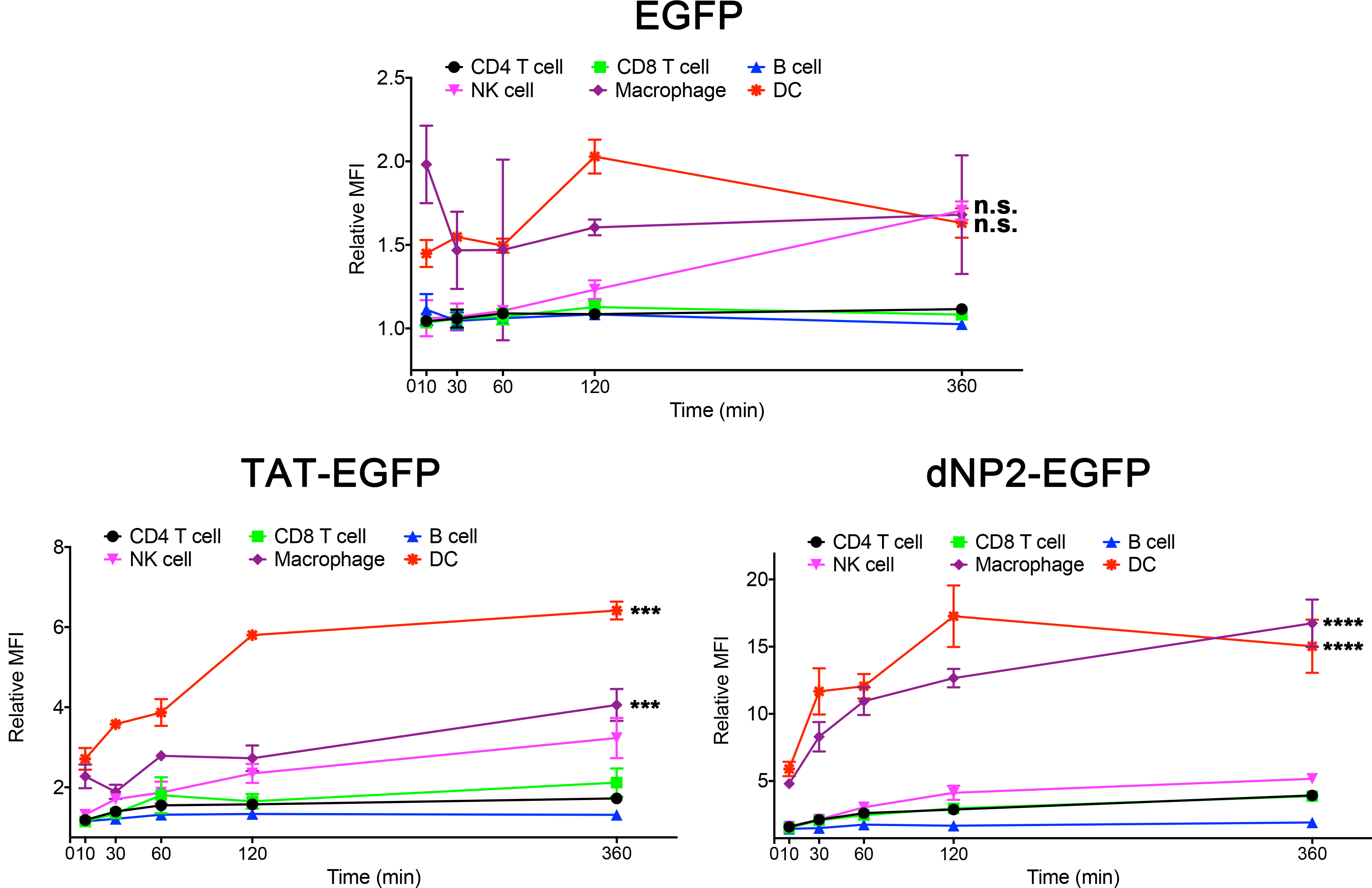

Supplement: S2 Fig — 5 μM of EGFP, TAT-EGFP, dNP2-EGFP or PBS were treated to splenocytes from 6-week-old C57BL/6 mice for various reaction times including 10 min, 30 min, 1 h, 2 h or 6 h and protein delivery efficiencies were analyzed by flow cytometry after cell staining with cell type specific markers (CD4+ CD4 T cells, CD19+ B cells, NK1.1+ NK cells, CD11clowCD11bhighF4/80+ macrophages, and MHCII+CD11chigh DCs). The values were normalized with the mean fluorescence intensity (MFI) of PBS treated samples (relative MFI). The graphs indicate the mean ± s.d. In the case of TAT-EGFP and dNP2-EGFP, macrophages and DCs showed significantly higher delivery efficiency than lymphocytes within every time points. We performed statistical analysis by two-way ANOVA with Tukey’s multiple comparisons test and *** indicates p<0.001 and **** indicates p<0.0001. (TIF) [file pone.0155689.s002.tif]

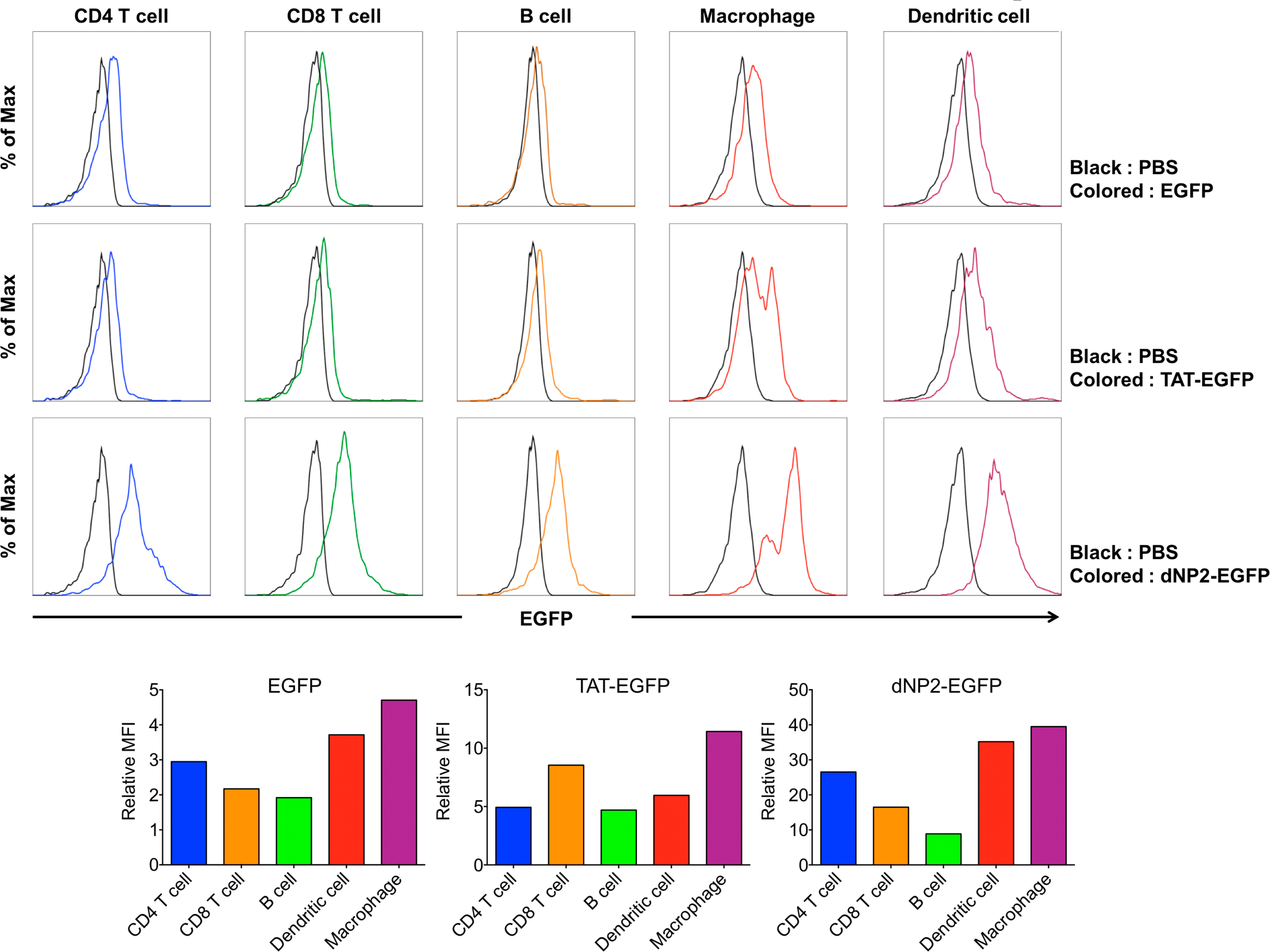

Supplement: S3 Fig — 5 μM of EGFP, TAT-EGFP, dNP2-EGFP or PBS were treated to FACS sorted CD4 T cells (CD4+), CD8 T cells (CD8+), B cells (CD19+), Dendritic cells (MHCII+CD11chigh) or Macrophages (CD11clowCD11bhighF4/80+). The delivery efficiencies were analyzed by flow cytometry. In the bar graphs, the values were normalized with MFI of PBS treated samples (relative MFI). (TIF) [file pone.0155689.s003.tif]
